# 

**Published:** 1900-12

**Authors:** 


### The Library of the Bristol Medico-Chirurgical Society.

*The following donations have been received since the publication of the List in September:*

*November 30th, 1900.*

| | |
|---|---|
| L. M. Griffiths (1) ... ... ... ... ... ... ... | 3 volumes. |
| Manchester Local Committee of the Library Association (2) ... ... ... ... ... ... ... | 1 volume. |
| The Council of the Royal College of Surgeons of England (3) ... ... ... ... ... ... ... | 1 ,, |
| The Council of University College, Bristol (4)... | 1 ,, |
| Charles Williams (5) ... ... ... ... ... ... | 6 volumes |

A framed picture has been presented by Mr. W. H. Harsant Unframed pictures have been received from the Editors of *The American Gynæcological and Obstetrical Journal* and *The Medical News.*

### THIRTY-EIGHTH LIST OF BOOKS.

The titles of books mentioned in previous lists are not repeated.

The figures in brackets refer to the figures after the names of the donors, and show by whom the volumes were presented. The books to which no such figures are attached have either been bought from the Library Fund or received through the *Journal.*

**Burghard, W. W. Cheyne and F. F.** *A Manual of Surgical Treatment.* Part IV. ... ... ... ... ... ... ... ... ... 1900
**Carless, W. Rose and A.** *Manual of Surgery* ... ... ... ... 3rd Ed. 1900
**Caton, R.** ... ... *The Temples and Ritual of Asklepios* ... ... 2nd Ed. 1900
**Cole, L. Riverius, N. Culpeper and A.** *The Practice of Physick* ... ... 1661
**Culpeper and A. Cole, L. Riverius, N.** *The Practice of Physick* ... ... 1661
**Cheyne and F. F. Burghard, W. W.** *A Manual of Surgical Treatment.* Part IV. ... ... ... ... ... ... ... ... ... 1900
**Duncan, J.** ... ... *Angioma, and other Papers.* (Ed. by J. Hodsdon) ... 1900
**Eade, Sir P.**... ... *The Norfolk and Norwich Hospital, 1770 to 1900* ... 1900
**Eshner, S. Solis-Cohen and A. A.** *Essentials of Diagnosis*... ... 2nd Ed. 1900
**Findlay, W.** ... *Robert Burns and the Medical Profession* ... ... ... 1898
**Gadd, H. W.** ... *A Synopsis of the British Pharmacopœia* ... 5th Ed. 1900

**Gould, G. M.** ... *The Student's Medical Dictionary* ... ... ...11th Ed. 1900
**Guttmann, H.** ... *Arzneiverordnungen in der Kinderpraxis.* Dritte Aufl. 1901
**Haddon, A. C.** ... *The Study of Man* ... ... ... ... ... ... ... ... ... 1898
**Harrison, R.** ... *Vasectomy and Urethro-Stenosis* ... ... ... ... ... 1900
**Kayser, R.** ... ... *Kehlkopf-, Nasen- und Ohrenkrankheiten* ... ... ... 1901
**Keetley, C. B.** ... *Orthopædic Surgery* ... ... ... ... ... ... ... ... ... 1900
**Keith, S.** ... ... *Gynæcological Operations* ... ... ... ... ... ... ... 1900
**Latimer, J.** ... ... *The Annals of Bristol in the Seventeenth, Eighteenth, and Nineteenth Centuries.* 3 vols. ... ... 1887–1900
**McCrae, W. Osler and T.** *Cancer of the Stomach* ... ... ... ... ... ... ... 1900
**Murrell, W.** ... *What to do in Cases of Poisoning* ... ... ... 9th Ed. 1900
**Osler and T. McCrae, W.** *Cancer of the Stomach* ... ... ... ... ... ... ... 1900
*Pharmacopœia in usum Nosocomii Regalis Bristoliensis* ... ... ... ... (1) 1858
**Prichard, J. C.** ... *Six Ethnographical Maps* ... ... ... ... 2nd Ed. 1861
**Purdy, C. W.** ... *Practical Uranalysis and Urinary Diagnosis* 5th Ed. 1900
**Quain, [J.]** ... ... *Elements of Anatomy.* (Ed. by E. A. Schäfer and G. D. Thane) Vol. I., part 1. New Ed. 1898
**Riolanus, J.** ... *Encheiridium Anatomicum et Pathologicum* ... ... ... 1649
**Riverius, N. Culpeper and A. Cole, L.** *The Practice of Physick* ... ... ... 1661
**Roberts, R. L.** ... *Nursing and Hygiene* ... ... ... ... ... 3rd Ed. 1900
**Rose and A. Carless, W.** *Manual of Surgery* ... ... ... ... ... 3rd Ed. 1900
*Royal College of Surgeons of England, Souvenir of the Centenary of the, 1800–1900* ... ... ... ... ... ... ... ... ... (3) 1900
**Seitz, C.** ... ... *Kurzgefasstes Lehrbuch der Kinderheilkunde.* Zweite Aufl. 1901
**Solis-Cohen and A. A. Eshner, S.** *Essentials of Diagnosis* ... ... 2nd Ed. 1900
**Stewart, G. N.** ... *Manual of Physiology* ... ... ... ... ... 4th Ed. 1900
**Sutton, C. W.** ... *Special Collections of Books in Lancashire and Cheshire* (2) 1900
**Thornton, R. J.** ... *The Pharmacopœia of the Royal College of Physicians of London.* (Tr. from Latin Ed., 1817) (1) 2nd Ed. 1817
**Todd, R. B.** ... ... *Clinical Lectures: Acute Diseases* ... ... ... ... (5) 1860
**Turner, W.** ... ... *Lectures on the Comparative Anatomy of the Placenta.* 1st Ser. (1) 1876
**Walsh, D.** ... ... *Golden Rules of Skin Practice* ... ... ... ... ... ... ... [1900]
**Williams, C.** ... *The Barber-Surgeons of Norwich* ... ... (5) 2nd Ed. 1897
,, ... *The Masters, Wardens, and Assistants of the Gild of Barber-Surgeons, 1439-1723.* 2nd Ser. ... (5) 1900
,, ... *The Ordinances of the Gild of Barber-Surgeons of Norwich* ... ... ... ... ... ... ... ... (5) 1900

### TRANSACTIONS, REPORTS, JOURNALS, &c.,

American Journal of Obstetrics, The ... ... ... ... ... Vol. XLI. 1900

American Ophthalmological Society, Transactions of the Vol. IX., pt. 1 1900

American Surgical Association, Transactions of the ... ... Vol. XVIII. 1900

Archives of Otology ... ... ... ... ... Vols. VIII., IX., 1879-80, XI. 1882

Australasian Medical Directory, The ... ... ... ... ... ... ... ... ... 1900

Bookseller, The ... ... ... ... ... ... ... ... ... ... ... ... ... ... 1899

British Library Year Book ... ... ... ... ... ... ... ... ... ... ... 1900

Buffalo Medical Journal ... ... ... ... ... ... ... ... ...Vol. XXXIX. 1900

Cincinnati Lancet-Clinic, The ... ... ... ... ... ... Vol. LXXXII. 1900

Clinical Journal, The ... ... ... ... ... ... ... ... ... Vol. XVI. 1900

Edinburgh Medical Journal, The ... ... ... ... ... ... Vol. VII. 1900

Edinburgh Obstetrical Society, The Transactions of the... Vol. XXV. 1900

Finska Läkaresällskapets sjuttonde allmänna möte i Helsingfors 21–23 September, 1899, Förhandlingar vid ... ... ... ... ... ... ... 1900
Giornale internazionale delle Scienze mediche ... ... ... ... ... ... 1899
Grant College Medical Society, Transactions of the, for 1899 ... ... 1900
Hospitals—
Edinburgh Hospital Reports ... ... ... ...Vols. V., VI. 1898–1900
Guy's Hospital Reports ... ... ... ... ... ... Vol. LIV. 1900
Journal of Tropical Medicine, The ... ... ... ... ... ... Vol. II. 1900
Library Association Record, The ... ... ... ... ... ... ... Vol. I. 1899
Library World, The ... ... ... ... ... ... ... ... ... ... Vol. II. 1900
Medical Society of London, Transactions of the ... ... Vol. XXIII. 1900
Medico-Chirurgical Transactions ... ... ... ... ... Vol. LXXXIII. 1900
New York, Transactions of the Medical Society of the State of ... ... 190
Ohio State Medical Society, Transactions of the ... ... ... ... ... 190
Philadelphia Medical Journal, The ... ... ... ... ... ... Vol. V. 1900
Polyclinic, The ... ... ... ... ... ... ... ... ...Vols. I., II. 1899–1900
University College, Bristol—Calendar ... ... ... ... ... ... ... (4) 1900
Western Ophthalmologic and Oto-Laryngologic Association, Transactions of the Ophthalmologic Division of ... ... ... ... ... ... 1900

---

### Local Medical Notes.

### BRISTOL.

University College.—C. G. Russ Wood, M.R.C.S., L.R.C.P., a former student of the School, has been elected Surgeon to the Eye, Ear, and Throat Hospital for Shropshire and Wales. There were seventeen competitors for the post.—A. G. G. Plumley, M.B. Lond., M.R.C.S., L.R.C.P., L.D.S., has been appointed Dental Surgeon to the Clayton Hospital and Wakefield General Dispensary.—Messrs. Dundas Edwards and J. Norton, of the Bristol Medical School, have obtained the M.R.C.S. Eng. and L.R.C.P. Lond. Diplomas.—A Rifle Club has been started at the College, and is to be connected with the National Rifle Association. There is a great difficulty in finding a range. In the meantime it is hoped that a Morris Tube range will be acquired in a situation near the College.

Royal Infirmary.—Alex. Ogilvy, M.D., F.R.C.S.I., has been appointed Ophthalmic Surgeon to the Royal Infirmary, in succession to F. Richardson Cross, M.B., F.R.C.S.—C. F. Walters, M.R.C.S., has been appointed Anæsthetist and Junior House Surgeon, and F. A. Coates, M.R.C.S., Junior House Surgeon, at the same Institution.

General Hospital.—H. G. Kyle, M.A., M.B., B.Ch. Oxon., has been appointed House Surgeon to the Hospital in succession to F. N. Cookson, M.D., F.R.C.S.

Memorial to the late Mr. Augustin Prichard.—On November 1st, at Redland Green Chapel, a window was dedicated to the memory of the late Mr. Augustin Prichard. The window contains the figures of St. Luke and St. Barnabas, and has a suitable inscription.

Scarlet Fever.—We are indebted to Dr. D. S. Davies for the following information with regard to the recent outbreaks of scarlet fever in Clifton:—At a meeting of the Health Committee on November 6th, the Medical Officer of Health reported an outbreak of scarlet fever in the registration sub-district of Clifton, which he ascribed to milk-infection. Sixty-three cases arose within five days in several parts of the sub-district which had previously been free, and all the cases were upon one or another of three milk rounds, all of which received part supply from one farm. Amongst 85 other milk rounds, supplying some 7000 houses, only 9 cases occurred during the same period. On enquiry at the suspected farm a boy was found to have suffered from indeterminate feverish and throat symptoms, and two of his brothers sickened of scarlet fever during the six days under notice: the first boy, considered as an ambulant case, had free access to the milk-cans during dairy operations. Several special instances pointing to the influence of uncooked milk in determining infection were quoted, and the Medical Officer pointed out that in residential districts any sudden and scattered outbreak of scarlet fever was almost certain to be due to milk carriage, and commented on the distinctions between the incidence in milk outbreaks and the slow gradual spread through the ordinary social channels. A full report of the outbreak will be published.

Medical School Annual Dinner.—On Thursday evening, Nov. 22nd, at the Royal Hotel, College Green, the past and present Bristol Medical Students held their Annual Dinner. It was a very successful function, and the medical profession of Bristol and Clifton were well represented. Dr. Shingleton Smith occupied the Chair, and the guest of the evening was Sir William Broadbent, Bart. After other toasts had been duly honoured, Sir William Broadbent proposed that of The Bristol Medical School. He felt that the chief right he had to propose this toast lay in the fact that he felt so much sympathy with it and that he wished well of the School. He himself had begun his medical career in the provinces, having attended classes at Owens College, Manchester. He had heard of the Bristol Medical School throughout his whole career. They were happy in having the School as part of University College, and he hoped it would not be long before it became a great Western University. He considered a Medical School a good nucleus for a University, and he hoped one day to see Bristol able to confer degrees in medicine. There was a very large field for clinical work open to the student at the Royal Infirmary, the General Hospital, the Eye, Children's, and Fever Hospitals, and the Asylum at Fishponds. It had given him great pleasure that afternoon to go over the local institutions and University College in which the preliminary sciences were taught. He did not recommend too much time to be spent over preliminary work, though he would not underrate the importance of anatomy and physiology. It was in hospital wards that the eye, ear, and hand were best trained, and here they learnt to deal with disease, not as an entity, but with the patient. Therefore everything must be subordinate to clinical work, and here Bristol had the advantage over some of the London Schools in the amount of material available *per* man; and thus he thought that Bristol might even attract students from London. Physicians and surgeons learnt as much in teaching as did the students. Expounding a case and going over notes helped exact observation, and he said this from an extended experience. He remembered hearing some students criticising a physician. One remarked, "He is never wrong;" the other said, "But he is never right either." Error, he thought, was better than confusion, and much was to be learnt from mistakes. He wished success to the faculty, the students, and the Medical School.

Dr. Markham Skerritt, the Dean of the Medical School, responded to the toast in suitable terms, and thanked Sir William Broadbent for coming there and speaking in such a kind way of University College, Bristol.

The health of the Chairman, Dr. Shingleton Smith, was proposed by Mr. R. W. Doyne, Oxford.

Dr. Smith's reply brought the meeting to a conclusion.

Royal Medical Benevolent College.—Mr. J. Paul Bush (the honorary secretary for Bristol) wishes to bring before the notice of the medical profession in this district the urgent need of new subscribers to the Royal Medical Benevolent College, the funds of which have suffered considerably owing to the numerous appeals in connection with the war in South Africa to which such splendid response has been given. That the good work is urgently needed is proved by the long list of applicants for "Pensionships" and "Foundation Scholarships." How well the Medical College at Epsom has done its duty to the latter class is shown by the success in after life of its "old boys." It has been with the utmost difficulty that the number of each class has been kept up to 50; it is increasingly clear that the Council must reduce the number of both Pensioners and Foundation Scholars if the income of the College is not maintained. Mr. Bush trusts that the profession of Bristol and the neighbourhood will do all that can be done to prevent any diminution of the funds of so useful a medical institution, and he hopes that the small number of only 24 subscribers for such a large and rich district will be considerably increased during the year.

### BATH.

Vaccination.—At the meeting of the Bath Board of Guardians, held on October 3rd, it was reported that during the first eight months of this year there had been 1,128 births, and of these children 658 had been successfully vaccinated, 99 cases were postponed, 67 had exemption certificates, and 154 had died.

### EXETER.

West of England Eye Infirmary.—The annual meeting of this institution was held on October 26th, under the presidency of the Rev. E. Chatterton Orpen. The medical report stated that 2,845 patients had been under treatment during the past year. The financial statement showed that the total receipts amounted to £1,392, and that there was a favourable balance of £86. The chairman stated that the first two sections of the new building were now nearly completed. He added that the cost of the new premises would be about £16,000, towards which £8,000 had been at present contributed.

### GLOUCESTER.

Health of Gloucestershire.—The report of the Medical Officer of Health and Sanitary Committee of the Gloucestershire County Council for 1899 shows that 8,802 births were registered (2,363 in urban and 6,439 in rural districts), giving a birth rate of 23.6 per 1,000 (against 29.3 for England and Wales); 5,458 deaths occurred (1,568 urban and 3,890 rural), equal to 14.97 per 1,000, as against 18.3 per 1,000 for England and Wales; 1,081 cases of scarlet fever were notified, with 18 deaths; 361 cases of diphtheria, with 61 deaths; 163 cases of typhoid fever, with 34 deaths; and 21 cases of puerperal fever, with 12 deaths. Only one case of small-pox was notified, and the report adds that "the Vaccination Act is working successfully, the domiciliary visits and the use of sterilised calf-lymph seem to have disarmed the opposition of objectors and the neglect of the indifferent."

### PLYMOUTH.

Ice-cream.—The borough analyst of Plymouth having reported to the Sanitary Committee of that town in reference to the ice-cream sold in the streets, the Sanitary Committee has decided that premises where ice-cream is manufactured shall be registered and supervised by the sanitary authority.

### RHONDDA VALLEY.

Scarlet Fever.—Owing to the prevalence of scarlet fever in the Rhondda Valley, the District Council has called upon the school authorities to close seven of the public elementary schools for a period of six weeks. The outbreak began about a fortnight after the schools re-assembled; the weekly number of cases since then has averaged nearly sixty.

### SWANSEA.

General Hospital.—Dr. F. G. Thomas and Mr. J. Thomas have been appointed Ophthalmic Surgeon and Consulting Surgeon respectively.

### WEYMOUTH.

Royal Hospital.—Dr. J. Moorhead has been appointed Consulting Physician.

---

### Obituary.

### LIST OF SUBSCRIBERS

TO

### The Bristol Medico-Chirurgical Journal.

*DECEMBER*, 1900.

---

### England.

### CAMBRIDGESHIRE.

CAMBRIDGE.

G. S. Woodhead

WALPOLE ST. ANDREW.

R. Smith

### CHESHIRE.

ALDERLEY EDGE.

J. McElfatrick

NEW BRIGHTON.

A. W. Riddell

### CORNWALL.

CAMBORNE.

T. C. Grey

HELSTON.

B. C. Kendall

LISKEARD.

S. E. Rigg

PENZANCE.

W. S. Bennett
J. A. Fox
J. Symons

ST. AUSTELL.

W. Mason

### DEVONSHIRE.

BABBACOMBE.

T. Finch

BAMPTON.

A. R. Down

BARNSTAPLE.

C. H. Gamble

J. Harper

M. Jackson

BRIXHAM.

G. C. Searle

BROAD CLYST.

J. Somer

BUDLEIGH SALTERTON.

T. G. C. Evans

CHAGFORD.

A. D. Hunt

CULLOMPTON.

G. G. Gidley

DARTMOUTH.

R. B. Searle

EXETER.

J. M. Ackland
H. Davy
R. V. Solly
E. B. Steele-Perkins
R. Thomas
L. H. Tosswill

NEWTON ABBOT.

J. Culross

### DEVONSHIRE *(Continued).*

PAIGNTON.

J. Alexander

PLYMOUTH.

G. F. Aldous
R. H. Lucy
Medical Society
E. B. Thomson
W. L. Woollcombe

PLYMPTON.

C. Aldridge
W. D Stamp

SOUTH MOLTON.

H. J. Smyth

TEIGNMOUTH.

T. E. Frazer-Toovey

TORQUAY.

W. Odell
R. Pollard
W. Powell
C. H. Wade

WINKLEIGH.

J. H. Norman

### DORSETSHIRE.

DORCHESTER.

P. W. MacDonald

WEYMOUTH.

J. M. Lawrie

### ESSEX.

NEWPORT.

W. A. Smith

WALTHAMSTOW.

F. P. Elliott

### GLOUCESTERSHIRE.

ALMONDSBURY.

J. C. McWatters

BRISTOL.

W. R. Ackland
J. Ambrose
W. M. Barclay
T. G. L'E Baretti
G. H. Barker
B. J. Baron
A. E. Blacker
A. F. Blagg
E. C. Board
C. W. J. Brasher
H. F. Briggs
F. St. J. Bullen
E. J. Bunbury
E. F. H. Burroughs
J. P. Bush
A. Carr
T. M. Carter
T. Carwardine
R. H. Chilton
J. M. Clarke
T. V. Coker
H. Cook
W. Cotton
R. J. Coulter
F. R. Cross
J. Dacre
D. S. Davies
N. C. Dobson
E. A. G. Dowling
E. L. W. Dunbar
E. Eberle
F. H. Edgeworth
W. Elder
C. Elliott
J. Ewens
E. Fawcett
R. G. Fendick
J. L. Firth
T. Fisher
E. L. Fox
J. Freeman
W. J. Fyffe
E. B. Garland
T. T. Genge
W. C. Gent
D. S Gerrish
A. N. G. Gibbs
J. S. Griffiths
L. M. Griffiths
C. D. G. Hailes
A. H. Haines
H. E. Harris
A. J. Harrison
W. H. Harsant
C. A. Hayman
Miss Heath
J. C. Heaven
C. K. C. Herapath
H. E. Hetling
H. Hill
T. Hill
General Hospital
G. A. Imlay
H. W. Kendall
F. P. Lansdown
R. G. P. Lansdown
A. E. A. Lawrence
E. L. Lees
R. C. Leonard
W. Ligertwood
J. J. S. Lucas
P. W. McCrea
A. R. McKee
G. Metcalfe
H. F. Mole
A. T. Morgan
C. A. Morton
G. T. Myles
W. N. Nevill
C. Newman
W. H. C. Newnham
T. D. Nicholson
N. Nield
C. O'Brien
A. Ogilvy
G. S. Page
G. Parker
J. H. Parry
A. W. Peake
W. A. Perry
C. F. Pickering
W. E. Pountney
J. J. Powell
A. W. Prichard
J. E. Prichard
A. B. Prowse

### GLOUCESTERSHIRE *(Continued).*

BRISTOL *(continued).*

D. C. Rayner
B. M. H. Rogers
F. H. Rose
W. B. Roué
C. K. Rudge
H. T. Rudge
J. E. Shaw
E. J. Sheppard
E. M. Skerritt
G. M. Smith
R. S. Smith
E. H. E. Stack
C. Steele
W. H. Steele
J. B. Stephenson
W. H. Stevens
S. V. Stock
F. W. Stoddart
J. Swain
J. G. Swayne
W. C. Swayne
J. O. Symes
J. Taylor
W. J. Tivy
H. Waldo
C. H. Walker
Canon Wallace
J. Wallace
J. H. Wathen
J. B. Webb
J. Wilding
E. C. Williams
P. W. Williams
W. R. Williams
W. K. Wills
H. W. Windsor-Aubrey
C. Wintle

CHELTENHAM.

W. J. K. Millard
G. A. Peake
E. Trevithick

CHIPPING SODBURY.

F. F. Fox
A. Grace

CIRENCESTER.

W. R. Cossham
O. H. Fowler
C. Mackinnon

DOWNEND.

H. Skelton

FISHPONDS.

H. A. Benham
C Bernard
W. Brown
A. L. Flemming

GLOUCESTER.

R. W. Batten
W. R. Hadwen
W. Hodges

HAMBROOK.

E. Crossman
F. W. Crossman
W. F. B. Eadon

KINGSWOOD.

E. W. H. Groves
L. W. Powell

LYDNEY.

W. P. Kennedy

REDFIELD.

J. W. Rodgers

ST. GEORGE.

J. Young

STOKE BISHOP.

H. F. Parsons.

STONEHOUSE.

G. T. B. Watters

STOW-ON-THE-WOLD.

E. Dening

STROUD.

A. S. Cooke

TETBURY.

W. Wickham

THORNBURY.

E. M. Grace
L. H. Williams

WESTBURY-ON-TRYM.

A. Harvey
H. L. Ormerod

WINTERBOURNE.

R. Eager
W. R. Eager

WOTTON-UNDER-EDGE.

J. G. Boyce
D. H. Forty

### HAMPSHIRE.

BOURNEMOUTH.

C. H. Ackland
J. S. Dickie
J. G. Harsant

CARISBROOKE.

J. Groves

FLEET.

H. Wilcox

LYMINGTON.

J. Rendall

SOUTHAMPTON.

W. R. Y. Ives

SOUTHSEA.

J. W. Cousins

### LANCASHIRE.

LIVERPOOL.

G. A. Hawkins-Ambler

MANCHESTER.

Bayer Co., Ltd.
Medical Society

### MIDDLESEX.

### LONDON.

J. Berry
T. C. Fox
M. Handfield-Jones
W. H. A. Jacobson
H. M. Jones
Lord Lister
E. J. Maclean
J. Macready
A. L. Marshall
F. T. Roberts
W. J. Seward
J. A. Shaw-Mackenzie

### TOTTENHAM.

W. H. Plaister

### TWICKENHAM.

A. E. Neale

### MONMOUTHSHIRE.

### NEWPORT.

W. Basset
C. B. Gratte
W. J. Greer
E. T. Hollings
O. E. B. Marsh
A. G. Thomas
H. E. Williams

### PONTYPOOL.

J. R. Essex
S. B. Mason

### RHYMNEY.

T. H. Redwood

### TREDEGAR.

G. A. Brown

### NOTTINGHAMSHIRE.

### NOTTINGHAM.

C. B. Taylor
W. M. Willis

### OXFORDSHIRE.

### OXFORD.

H. P. Symonds

### SOMERSETSHIRE

### BATH.

W. D. Akers
E. J. Cave
F. S. Cowan
S. Craddock
W. M. Ellis
T. B. Goss
F. Lace
T. P. Lowe
G. J. K. Martin
W. S. Melsome
R. J. H. Scott
L. H. Walsh
L. A. Weatherly
J. Wigmore
A. S. Wohlmann

### BRIDGWATER.

F. J. C. Parsons
J. L. K. Pringle
R. H. F. Routh

### BRISLINGTON.

B. B. Fox
W. B. Morton
C. M. Phillips

### BURNHAM.

F. C. Berry

### CHEDDAR.

E. H. Openshaw
R. W. Statham

### CLEVEDON.

T. Davis
W. J. Hill
J. Martin

### COMPTON MARTIN.

H. C. Linden

### CREWKERNE.

W. W. Webber

### CURRY RIVEL.

R. H. Vereker

### FRESHFORD.

C. E. S. Flemming

### SOMERSETSHIRE *(Continued).*

FROME.

A. W. Dalby
A. G. Hayman
J. M. Rattray
C. R. Wood

GLASTONBURY.

J. C. Smyth

HAM GREEN.

F. P. Mackie

ILMINSTER.

C. Munden

KEYNSHAM.

G. G. D. Willett

LANGPORT.

J. Morgan

MILVERTON.

C. Randolph

PILL.

T. W. S. Morgan

PORTISHEAD.

G. S. J. Boyd
W. Monckton
C. A. Wigan

TAUNTON.

H. T. S. Aveline
Taunton and Somerset Hospital
H. T. Rutherford

WEDMORE.

J. Ford
R. P. Tyley

WELLINGTON.

J. Meredith

WELLS.

W. Fairbanks
W. A. L. Smith
C. J. Whitby

WEST COKER.

G. C. Wilkin

WESTON-SUPER-MARE.

H. T. M. Alford
H. S. Ballance
C. P. Crouch
E. F. Martin
G. F. Rossiter
R. Roxburgh
G. H. Temple
J. Wallace

WORLE.

F. St. J. Kemm

WRINGTON.

H. C. Bristowe
H. W. Collins

YEOVIL.

W. R. M. Semple

### WARWICKSHIRE.

LEAMINGTON.

A. W. W. Hoffman

### WILTSHIRE.

BRADFORD-ON-AVON.

W. J. A. Adye
J. Beddoe

CALNE.

W. S. Batten

FOVANT.

C. Clay

MALMESBURY.

R. Kinneir

SWINDON.

J. C. Maclean

WARMINSTER.

R. L. Willcox

### WORCESTERSHIRE.

WORCESTER.

R. W. Brimacombe

### Ireland.

### ANTRIM.

### BELFAST.

J. W. Browne
A. Jamison
J. A. Lindsay

### Wales.

### BRECONSHIRE.

### BRECON.

W. Howells

### CARMARTHENSHIRE.

### FERRYSIDE.

P. Williams

### LLANELLY.

S. J. Roderick

### GLAMORGANSHIRE.

### ABERDARE.

E. Jones

### CARDIFF.

W. J. Corrigan
P. R. Griffiths
T. G. Horder
D. R. Paterson
A. Sheen
Medical Society
J. L. Thomas
H. R. Vachell
J. Williams

### GORSEINON.

T. Mitchell

### MERTHYR TYDVIL.

W. W. Jones
J. L. W. Ward

### PENARTH.

C. E. Matthews

### SWANSEA.

E. Le C. Lancaster

### TREHARRIS.

W. W. Leigh

### PEMBROKESHIRE.

### HAVERFORDWEST.

E. P. Phillips

### France.

### PARIS.

H. Le Soudier

### Switzerland.

### DAVOS-PLATZ.

W. R. Huggard

### Cape Colony.

### FORT BEAUFORT.

J. Shaw

### KING WILLIAM'S TOWN.

H. M. Chute

### United States.

### CHICAGO.

Columbus Medical Library

### COLORADO.

S. E. Solly

### PHILADELPHIA.

Philadelphia Medical Journal.

### Italy.

### GENOA.

S. Biamonti

### Egypt.

### CAIRO.

M. A. Ruffer

### New Zealand.

### LOWER HUTT.

J. R. Purdy

### India.

### BHAGALPORE.

N. Muzumidar

### CALCUTTA.

Major Peck

### INDEX.

Abdominal cases presenting some unusual features, Three—A. W. Prichard, 313.
Abdominal incisions — T. Carwardine, 204.
Abdominal operations, Retrospect of fifty intra—Dr. J. Swain, 103.
Albuminuria and Bright's disease—Dr. N. Tirard (*Review*), 158.
Albuminuria in the apparently healthy, 35.
Alchemy and pharmacy, The mystery and romance of — C. J. Thompson (*Review*), 48.
Alcohol, The *rôle* of — Dr. E. M. Cosgrave (*Review*), 263.
Alimentary canal, Surgery of malignant disease in the, 146.
Alimentary canal, Surgery of the—Dr. A. E. Maylard (*Review*), 256.
Allbutt, Dr. T. C. [Ed.]—A system of medicine (*Review*), 51.
American Association of Obstetricians and Gynecologists, Transactions of the (*Review*), 267.
American Dermatological Association, Transactions of the (*Review*), 372.
American Laryngological Association, Transactions of the (*Review*), 267.
American medical quarterly, The (*Review*), 375.
American Ophthalmological Society, Transactions of the (*Review*), 267.
American Pediatric Society, Transactions of the (*Review*), 268.
American Physicians, Transactions of the Association of (*Review*), 376.
American Public Health Association, Reports and papers of the (*Review*), 170.
American Surgical Association, Transactions of the (*Review*), 268.
American year-book of medicine and surgery, The (*Review*), 373.
Anæmia—Dr. B. Bramwell (*Review*), 55.
Anæsthesia, One thousand inductions of—C. H. Whiteford, 217.
Anæsthetics — Dr. D. W. Buxton (*Review*), 249.
Anatomy of the central nervous system of man—Dr. L. Edinger (*Review*), 242.
Anderson, Dr. J. W.—The power of nature in disease (*Review*), 262.
Animal simples—Dr. W. T. Fernie (*Review*), 64.
Annual and analytical cyclopædia of practical medicine (*Reviews*), 69, 377.
Appendicitis, Forty cases of operation for—C. A. Morton, 320.
Ascites of cirrhosis, The surgical treatment of the, 38.
Aseptic surgery—C. B. Lockwood (*Review*), 256.
Australia, On the uses and abuses of the public hospitals in — L. Bruck (*Review*), 274.

Bannatyne, Dr. G. A. — The thermal waters of Bath (*Review*), 167.
Baron, Dr. B. J.—Report on laryngology and rhinology, 348.
Bath, The thermal waters of—Dr. G. A. Bannatyne (*Review*), 167.
Bell, Dr. R.—The pathogenesis and treatment of cancer without operation (*Review*), 366.
Bibliographia medica (*Review*), 166.
Bramwell, Dr. B. — Anæmia and some of the diseases of the blood-forming organs and ductless glands (*Review*), 55.
Breast, Halsted's operation for cancer of the—C. H. Whiteford, 336.
Breast, Oöphorectomy in cancer of the, 345.
Bright's disease, Albuminuria and—Dr. N. Tirard (*Review*), 158.
Bristol Medico-Chirurgical Society, 75, 175, 365.
Bristol Royal Infirmary, Reminiscences of the—A. W. Prichard, 193.
Broadbent, Sir W. H. and Dr. J. F. H.—Heart disease (*Review*), 159.
Brockbank, Dr. E. M.—The murmurs of mitral disease (*Review*), 255.
Broom, Obituary notice of Dr. John, 186.
Browne, L.—The throat and nose, and their diseases (*Review*), 164.
Bruce, Dr. J. M.—Materia medica and therapeutics (*Review*), 260; The principles of treatment (*Review*), 356.
Bruck, L.—On the uses and abuses of the public hospitals in Australia, Tasmania, and New Zealand (*Review*), 274.
Burdon-Sanderson, J.—Ludwig and modern physiology (*Review*), 250.

Burghard, Drs. W. W. Cheyne and F. F.—A manual of surgical treatment (*Review*), 247.
Burns produced by carbolic acid, Treatment of, 236.
Buxton, Dr. D. W.—Anæsthetics: their uses and administration (*Review*), 249.
Buxton: its history, waters, climate, scenery, &c. (*Review*), 261.

Cancer—W. R. Williams (*Review*), 165.
Cancer without operation, The pathogenesis and treatment of—Dr. R. Bell (*Review*), 366.
Carbolic gangrene, 343.
Cardiac muscle, Rheumatic disease of the—Dr. T. Fisher, 16.
Carless, W. Rose and A.—A manual of surgery (*Review*), 367.
Carter, Obituary notice of Mr. W. F., 186.
Caruncle of the urethra, Vascular—Dr. A. E. A. Lawrence, 23.
Carwardine, T.—Abdominal incisions, 204; Report on surgery, 236.
Cerebro-spinal fluid, The—Dr. StC. Thomson (*Review*), 162.
Cheadle, Dr. W. B.—Occasional lectures on the practice of medicine (*Review*), 358.
Chemistry, A manual of—Dr. A. P. Luff and F. J. M. Page (*Review*), 60.
Chemistry, A text-book of physical—Dr. R. A. Lehfeldt (*Review*), 155.
Chest, Thoracic resection for tumors growing from the bony wall of the—Dr. F. W. Parham (*Review*), 59.
Cheyne and F. F. Burghard, Drs. W. W.—A manual of surgical treatment (*Review*), 247.
Children, Diseases of—Drs. G. Elder and J. S. Fowler (*Review*), 68; Dr. J. McCaw (*Review*), 256.
Children, Experimental studies of—A. MacDonald (*Review*), 67.
Children, Growing—E. N. Smith (*Review*), 169.
Children, Mentally-deficient—Dr. G. E. Shuttleworth (*Review*), 368.
Christofferson, E.—Swedish medical gymnastics in chronic diseases of the heart, 134.
Cirrhosis, Surgical treatment of the ascites of, 38.
Clarke, Dr. J. J.—Orthopædic surgery (*Review*), 248.
Clarke, Dr. J. M.—Report on medicine, 338; Two cases of the sporadic form of epidemic cerebrospinal meningitis, 128.
Clinical Society of London, Transactions of the (*Review*), 264.
Coats, Dr. J.—Manual of pathology (*Review*), 368.
Coblentz, Dr. V.—The newer remedies (*Review*), 260.
Cœliotomy, Fifty consecutive cases of—Dr. J. M. Lawrie (*Review*), 258.
Cokenower, Dr. J. W.—Contributions of original articles to orthopedic surgery (*Review*), 257.
Conjunctivitis, 44.
Constipation—Dr. G. Herschell (*Review*), 168.
Consumption—Dr. W. H. Spencer (*Review*), 254.
Consumption chart and case paper (*Review*), 375.
Consumption, Open-air treatment of—Dr. Jane H. Walker (*Review*), 169.
Consumptives, Sanatoria for the poorer—Dr. L. A. Weatherly, 305.
Copeman, Dr. S. M.—Vaccination: its natural history and pathology (*Review*), 65.
Cosgrave, Dr. E. M.—Experimental proofs of the *rôle* of alcohol (*Review*), 263.
Cotton, Dr. W.—The inebriates' acts, 1879-1899, 222.
Cremation, Modern—Sir H. Thompson (*Review*), 66.
Creuznach-Spa and its environs (*Review*), 262.
Cross, F. R.—Report on ophthalmology, 41.
Croup, Non-bacillary—Dr. J. O. Symes, 25.

Davies, Dr. D. S.—Some modern aspects of preventive medicine, 289.
Deformities of the lower extremities, Paralytic—E. N. Smith (*Review*), 359.
Dermatitis multiformis exfoliativa—Dr. W. Spencer (*Review*), 371.
Dermatological Society of Great Britain and Ireland, Transactions of the (*Review*), 267.
Dermatology, An introduction to—Dr. N. Walker (*Review*), 163.
Dermatology, Report on—Dr. H Waldo, 240.
Diabetes mellitus, Klinische erfahrungen über—Dr. E. Külz [*et al.*] (*Review*), 57.
Diagnosis, Differential.—Dr. F. J. Smith (*Review*), 244.
Dictionary, A pocket medical—Dr. G. M. Gould (*Review*), 252.
Dictionnaire des termes de médecine, français-anglais—H. de Méric (*Review*), 49.

Diet in typhoid fever, 33.
Dietetic treatment of zymotic enteritis—Dr. F. P. Elliott, 225.
Digest, The medical—Dr. R. Neale (*Review*), 274.
Digestion due to displacements, Difficult—Dr. A. S. Eccles (*Review*), 57.
Directory of laryngologists and otologists, International (*Review*), 376.
Disease, The power of nature in—Dr. J. W. Anderson (*Review*), 262.
Dislocations of the upper extremity, Fractures and—J. E. Platt (*Review*), 367.
Displacements, Difficult digestion due to—Dr. A. S. Eccles (*Review*), 57.
Drugs, Analysis of—T. H. Pearmain and C. G. Moor (*Review*), 261.
Dührssen, Dr. A.—A manual of gynæcological practice (*Review*), 258.
Duodenum, Ulcer of the stomach and—Drs. S. and W. S. Fenwick (*Review*), 357.

Eastbourne as a health resort—C. Roberts (*Review*), 262.
Eccles, Dr. A. S.—Difficult digestion due to displacements (*Review*), 57.
Eclampsia, Lectures on hæmorrhage and—Dr. R. Jardine (*Review*), 369.
Eczema—Dr. T. Robinson (*Review*), 370.
Edinburgh hospital reports (*Review*), 375
Edinburgh, Laboratory reports of the Royal College of Physicians of (*Review*), 376.
Edinburgh medical journal (*Review*), 273.
Edinger, Dr. L.—The anatomy of the central nervous system of man, and of vertebrates in general (*Review*), 242.
Elder and J. S. Fowler, Drs. G.—The diseases of children (*Review*), 68.
Electricity, Therapeutic—Dr. W. S. Hedley (*Review*), 64.
Elliott, Dr. F. P.—The dietetic treatment of zymotic enteritis, especially with regard to the use of raw meat juice, 225.
Ellis, H.—Studies in the psychology of sex (*Review*), 50.
Emond, Dr. E.—Le Mont-Dore et ses eaux minèrales (*Review*), 261.
Emotions, The pathology of—C. Féré (*Review*), 363.
Encyclopædia medica—Dr. C. Watson [Ed.], (*Reviews*), 55, 156.
Enlargement of the prostate—Dr. C. M. Moullin (*Review*), 367.
Enteritis, The dietetic treatment of zymotic—Dr. F. P. Elliott, 225.
Epidemiological Society of London, Transactions of the (*Review*), 170.
Evans, Dr. A. H.—Golden rules of medical practice (*Review*), 253.
Examinations, Guide to—F. J. Gant (*Review*), 263.
Exploration of the urethra and bladder—M. Tuchmann (*Review*), 63.
Extremities, Paralytic deformities of the lower—E. N. Smith (*Review*), 359.
Eye accidents occurring in trades—S. Snell (*Review*), 369.
Eye, Atlas of external diseases of the —Dr. A. M. Ramsay (*Review*), 360.

Faucitis, Recurring membranous, 348.
Fayrer, Sir J.—Recollections of my life (*Review*), 353.
Fenwick, Drs. S. and W. S.—Ulcer of the stomach and duodenum (*Review*), 357.
Féré, C.—The pathology of emotions (*Review*), 363.
Fernie, Dr. W. T.—Animal simples approved for modern uses of cure (*Review*), 64.
Fisher, Dr. T.—Rheumatic disease of the cardiac muscle, 16; Report on medicine, 33.
Fitzgerald, J.—Pyorrhœa alveolaris (*Review*), 259.
Food, Analysis of—T. H. Pearmain and C. G. Moor (*Review*), 261.
Foods, Flesh—C. A. Mitchell (*Review*), 359.
Foster, M.—Recent advances in science, and their bearing on medicine and surgery (*Review*), 251; Recent progress in physiology (*Review*), 251.
Fothergill, Dr. W. E.—Golden rules of obstetric practice (*Review*), 369.
Fowler, Drs. G. Elder and J. S.—The diseases of children (*Review*), 68.
Fracture, The X-ray history of a—J. Taylor, 214.
Fractures and dislocations of the upper extremity—J. E. Platt (*Review*), 367.
Fractures and dislocations, Skiagraphic atlas of—Dr. D. J. Mackintosh (*Review*), 68.
Freeman, Capt. E. C.—The Sanitation of British troops in India (*Review*), 167.

Gadd, H. W.—A synopsis of the British Pharmacopœia, 1898 (*Review*), 351.

Gage, S. H.—The processes of life revealed by the microscope (*Review*), 252.
Gall-bladder and bile-ducts, Diseases of the—A.W. M. Robson (*Review*), 257.
Gangrene, Carbolic, 343.
Gant, F. J.—A guide to the examinations by the Conjoint Examining Board in England, and for the diploma of Fellow of the Royal College of Surgeons of England (*Review*), 263.
Gastric methods, A manual of modern — Dr. A. L. Gillespie (*Review*), 246.
Gillespie, Dr. A. L.—A manual of modern gastric methods, chemical, physical, and therapeutical (*Review*), 246.
Glasgow hospital reports (*Review*) 271
Gould, Dr. G. M.—A pocket medical dictionary (*Review*), 252.
Gout, 234.
Grant College Medical Society, Transactions of the (*Review*), 266.
Greer, W. J.— Spindle-celled sarcoma of the hard palate, 211.
Griffiths, Presentation to Mr. L. M., 97, 282.
Griffiths, W. H.—Lessons on prescriptions and the art of prescribing (*Review*), 170.
Groves, Dr. E. W. H.—Five cases of pelvic disease treated by laparotomy, 330.
Guy's hospital reports (*Review*), 269.
Gynæcological practice — Dr. A. Dührssen (*Review*), 258.

Hæmorrhage and eclampsia—Dr. R. Jardine (*Review*), 369.
Halliburton, Dr. W. D.–Kirkes' handbook of physiology (*Review*), 354.
Halsted's operation for cancer of the breast—C. H. Whiteford, 336.
Harding, Dr. W.—Mental nursing (*Review*), 71.
Harrison, R.— Selected papers on stone, prostate, and other urinary disorders (*Review*), 161.
Harrogate, The natural waters of—Dr. F. W. Smith (*Review*), 262.
Harsant, W. H.—Cases of acute intestinal obstruction, treated by operation, 118; Report on otology, 350.
Hartridge, G. — Golden rules of ophthalmic practice (*Review*), 368.
Hawkins-Ambler, G. A.—Gynæcological nursing (*Review*), 258.
Hawthorne, Dr. C. O. —Rheumatism, rheumatoid arthritis, and subcutaneous nodules (*Review*), 366.
Hawthorne, Dr. C. O.—Some aspects of the mental discipline associated with the study of medicine (*Review*), 252.
Health abroad—Dr. E. Hobhouse [Ed.] (*Review*), 364.
Heart disease—Sir W. H. and Dr. J. F. H. Broadbent (*Review*), 159.
Heart, On the principles which govern treatment in diseases and disorders of the (*Review*), 157.
Heart, The Schott methods of the treatment of chronic diseases of the—Dr. W. B. Thorne (*Review*), 255.
Hedley, Dr. W. S. — Therapeutic electricity and practical muscle testing (*Review*), 64.
Hernia, Treatment of inguinal, 347.
Herschell, Dr. G.—Constipation and its modern treatment (*Review*), 168.
Hillemand, Dr. C.—Organothérapie ou opothérapie (*Review*), 169.
Hillier, Dr. A.—Tuberculosis: its nature, prevention, and treatment, with special reference to the open air treatment of phthisis (*Review*), 253.
Hime, Dr. M. C. — Schoolboys' special immorality (*Review*), 263.
Hinshelwood, Dr. J.—Letter-, word- and mind-blindness (*Review*), 359.
Hobhouse, Dr. E. [Ed.]—Health abroad (*Review*), 364.
Hospitals and charities, Burdett's (*Review*), 376.
Hudson-Cox and Dr. J. Stokes, F.—A pocket pharmacopœia (*Review*), 260.
Humane review, The (*Review*), 273.
Hygiene and public health — Dr. B. A. Whitelegge (*Review*), 169.
Hydrorrhœa, Treatment of nasal, 350.
Hysterectomy for fibroids of the uterus, Abdominal — Dr. W. C. Swayne, 123.

Index-catalogue of the library of the Surgeon-General's Office, United States Army (*Review*), 274
India, The sanitation of British troops in — Captain E. C. Freeman (*Review*), 167.
Inebriates' acts, 1879-1899, The—Dr. W. Cotton, 222.
Infectious and contagious diseases in schools, Prevention of, (*Review*), 168.
Insanity, Vice and—Dr. G. R. Wilson (*Review*), 245.
Intestinal obstruction—W. H. Harsant, 118; F. Treves (*Review*), 256.
Iowa State Medical Society, Transactions of the (*Review*), 265.

Ireland, Transactions of the Royal Academy of Medicine in (*Review*), 265.
Irvine, Obituary notice of Lieut. G. H., 186.

Jardine, Dr. R. — Lectures on hæmorrhage and eclampsia (*Review*), 369; Practical text-book of midwifery for nurses and students (*Review*), 361.
Jenner Institute of Preventive Medicine, Transactions of the (*Review*), 166.
Jessop, W. H. H. — Manual of ophthalmic surgery and medicine (*Review*), 361.
Johns Hopkins hospital reports (*Reviews*), 70, 271.
Journal of surgical technology (*Review*), 377.
Journal of the American Medical Association, General index to the (*Review*), 374.

Kidney, Movable, 236.
Kinderheilkunde, Kurzgefasstes lehrbuch der—Dr. C. Seitz (*Review*), 362.
King's College hospital reports (*Review*), 269.
Kirkes' handbook of physiology—Dr. W. D. Halliburton (*Review*), 354.
Knee-joint, Internal derangement of the, 36.
Külz, Dr. E. [*et al.*] — Klinische erfahrungen über diabetes mellitus (*Review*), 57.

Laparotomy, Pelvic disease treated by—Dr. E. W. H. Groves, 330.
Laryngeal cancer, 348.
Laryngologists and otologists, International directory of (*Review*), 360.
Laryngology, Report on—Dr. B. J. Baron, 348.
Lawrence, Dr. A. E. A.—Vascular caruncle of the urethra, 23.
Lawrie, Dr. J. M.—Notes of fifty consecutive cases of cœliotomy for diseases of the uterus and appendages, with forty-eight recoveries (*Review*), 258.
Lehfeldt, Dr. R. A.—A text-book of physical chemistry (*Review*), 155.
Leontiasis ossea—Dr. E. H. E. Stack, 316.
Leser, Dr. E. — Operations - vademecum für den praktischen arzt (*Review*), 64.
Letter-, word- and mind-blindness —Dr. J. Hinshelwood (*Review*), 359.
Library of the Bristol Medico-Chirurgical Society, 85, 182, 277, 385.
Light treatment in lupus, 240.
Lister and surgery, Lord—Dr. R. Turner (*Review*), 250.
Local medical notes, 88, 186, 282, 387.
Lockwood, C. B.—Aseptic surgery (*Review*), 256.
London, How surgery became a profession in (*Review*), 250.
London, Transactions of the Clinical Society of (*Review*), 264.
London, Transactions of the Epidemiological Society of (*Review*), 170.
London, Transactions of the Medical Society of (*Review*), 170.
Lowestoft as a health resort (*Review*), 368.
Lucas, J. J. S.—Nordrach at home; or, hygienic treatment of consumption adapted to English home life (*Review*), 254.
Ludwig and modern physiology—J. Burdon-Sanderson (*Review*), 250.
Luff and F. J. M. Page, Dr. A. P.—A manual of chemistry (*Review*), 60.
Lupus, The light treatment in, 240.

McCaw, Dr. J.—Aids to the diagnosis and treatment of diseases of children (*Review*), 258.
MacDonald, A.—Experimental study of children (*Review*), 67.
Mackintosh, Dr. D. J.—Skiagraphic atlas of fractures and dislocations, with notes on treatment (*Review*), 68
Macpherson, Dr. J.—Mental affections: an introduction to the study of insanity (*Review*), 160.
Malaria, Notes on—Major E. M. Wilson (*Review*), 255.
Malignant disease in the alimentary canal, Surgery of, 146.
Mankind, The history of—F. Ratzel (*Review*), 45.
Manson, Dr. P.—Tropical diseases (*Review*), 365.
Marsh, Obituary notice of Major T. A. P., 185.
Materia medica and therapeutics—Dr. J. M. Bruce (*Review*), 260.
Materia medica, Merck's manual of the (*Review*), 170.
Maylard, Dr. A. E.—The student's handbook of the surgery of the alimentary canal (*Review*), 256.
Measles, Note on a pre-exanthematous sign of—Dr. P. W Williams, 139.
Medical annual (*Review*), 264.
Medical annual synoptical index to remedies and diseases (*Review*), 375.

Medical practice, Golden rules of—Dr. A. H. Evans (*Review*), 253.
Medical review, The (*Review*), 70.
Medical Society of London, Transactions of the (*Review*), 170.
Medicine, A system of—Dr. T. C. Allbutt [Ed.] (*Review*), 51.
Medicine, History of—Dr. R. Park (*Review*), 250.
Medicine, Lectures on clinical—Dr. J. L. Steven (*Review*), 253.
Medicine, Occasional lectures on the practice of — Dr. W. B. Cheadle (*Review*), 358.
Medicine, Reports on—Dr. J. M. Clarke, 338; Dr. T. Fisher, 33; Dr. G. Parker, 229; Dr. R. S. Smith, 141.
Medicine, Some aspects of the mental discipline associated with the study of—Dr. C. O. Hawthorne (*Review*), 252.
Medico - chirurgical transactions (*Review*), 171.
Meningitis, Two cases of the sporadic form of epidemic cerebro-spinal—Dr. J. M. Clarke, 128.
Mental affections—Dr. J. Macpherson (*Review*), 160.
Mentally-deficient children—Dr. G. E. Shuttleworth (*Review*), 368.
Merck's annual report (*Review*), 273; Manual of the materia medica (*Review*), 170.
Méric, H. de — Dictionnaire des termes de médecine, français-anglais (*Review*), 49.
Mexico—Informes rendidos por los inspectores sanitarios de cuartel y los de los distritos al consejo superior de salubridad (*Review*), 272.
Michigan State Medical Society, Transactions of the (*Review*), 265.
Microscope, The processes of life revealed by the — S. H. Gage (*Review*), 252.
Middlesex hospital reports (*Review*), 269.
Midwifery, Practical text-book of—Dr. R. Jardine (*Review*), 361.
Miles, Dr. A.—Surgical ward work and nursing (*Review*), 371.
Milk, The Pasteurisation of — Dr. J. O. Symes, 275.
Missions, Medical—G. E. F. M. (*Review*), 372.
Mitchell, C. A.—Flesh foods (*Review*), 359.
Mitral disease, The murmurs of—Dr. E. M. Brockbank (*Review*), 255.
Monro, Dr. T. K. — Raynaud's disease (*Review*), 247.
Mont-Dore et ses eaux minérales, Le—Dr. E. Emond (*Review*), 261.
Moor, T. H. Pearmain and C. G.—Aids to the analysis of food and drugs (*Review*), 261.
Morison, Dr. A.—On the relation of the nervous system to disease and disorder in the viscera (*Review*), 54.
Morton, C. A.—Forty cases of operation for appendicitis, 320; Report on surgery, 146.
Mott, Dr. F. W. [Ed.]—Archives of neurology from the pathological laboratory of the London County Asylums, Claybury, Essex (*Review*), 66.
Moullin, Dr. C. M.—Enlargement of the prostate (*Review*), 367.
Moure, Dr. E. J.—De la trachéo-thyrotomie dans le cancer du larynx (*Review*), 258.
Mouth, The hygiene of the—R. D. Pedley (*Review*), 259.
Mracek, Dr. F.—Atlas of syphilis and the venereal diseases (*Review*), 60.
Murray, Dr. W.—Rough notes on remedies (*Review*), 260.
Myasthenia gravis, 338
Myopia, 41.

Nature, Human: its principles and the principles of physiognomy—"Physicist" (*Review*), 366.
Neale, Dr. R.—The medical digest, or busy practitioner's vade-mecum (*Review*), 274.
Nervous system, Diseases of the—Dr. H. C. Thomson (*Review*), 245.
Nervous system of man, The anatomy of the central — Dr. L. Edinger (*Review*), 242.
Nervous system to disease and disorder in the viscera, On the relation of the — Dr. A. Morison (*Review*), 54.
Neurasthenia — Dr. T. D. Savill (*Review*), 168.
Neurology, Archives of—Dr. F. W. Mott [Ed.] (*Review*), 66.
Newman, Dr. D.—Renal cases (*Review*), 358.
Newsholme, Dr. A.—The elements of vital statistics (*Review*), 71.
New York, Transactions of the Medical Society of the State of (*Reviews*), 265, 373.
Nisbet, Drs. W. W. van Valzah and J. D.—The diseases of the stomach (*Review*), 58.
Nordrach at home—J. J. S. Lucas (*Review*), 254.
Nose and their diseases, The throat and—L. Browne (*Review*), 164.

Nose, Treatment of fractures of the, 348.
Nurses, A handbook for—Dr. J. K. Watson (*Review*), 162.
Nurses, Pocket case book for (*Review*), 169.
Nursing directory, Burdett's official (*Review*), 272.
Nursing, Gynæcological—G.A.Hawkins-Ambler (*Review*), 258.
Nursing in diseases of the throat, nose and ear—M. Yearsley (*Review*), 71.
Nursing, Mental—Dr. W. Harding (*Review*), 71.
Nursing, Surgical ward work and—Dr. A. Miles (*Review*), 371.

Obstetric practice, Golden rules of—Dr. W. E. Fothergill (*Review*), 369.
Obstetrics, Report on—Dr. W. C. Swayne, 151.
Ohio State Medical Society, Transactions of the (*Review*), 265.
Oöphorectomy in cancer of the breast, 345.
Operations - vademecum für den praktischen arzt—Dr. E. Leser (*Review*), 64.
Ophthalmic practice, Golden rules of—G. Hartridge (*Review*), 368.
Ophthalmic surgery and medicine, Manual of—W. H. H. Jessop (*Review*), 361.
Ophthalmology, Report on—F. R. Cross, 41.
Organothérapie ou opothérapie—Dr. C. Hillemand (*Review*), 169.
Orthopædic surgery—Dr. J. J. Clarke (*Review*), 248; Dr. J. W. Cokenower (*Review*), 257.
Otitis media, On opening the mastoid in chronic suppurative, 350.
Otologists, International directory of laryngologists and (*Review*), 360.
Otology, Report on—W. H. Harsant, 350.

Page, Dr. A. P. Luff and F. J. M.—A manual of chemistry (*Review*), 60.
Palate, Spindle-celled sarcoma of the hard—W. J. Greer, 211.
Parham, Dr. F. W.—Thoracic resection for tumors growing from the bony wall of the chest (*Review*), 59.
Park, Dr. R.—An epitome of the history of medicine (*Review*), 250.
Parker, Dr. G.—Report on medicine, 229.
Pasteurisation of milk, The — Dr. J. O. Symes, 275.
Pathology, A manual of—Dr. J. Coats (*Review*); 368.
Pearmain and C. G. Moor, T. H.—Aids to the analysis of food and drugs (*Review*), 261.
Pedley, R. D.—The hygiene of the mouth (*Review*), 259.
Pelvic disease treated by laparotomy—Dr. E. W. H. Groves, 330.
Pharmacopœia, 1898, A synopsis of the British — H. W. Gadd (*Review*), 367.
Pharmacopœia, The pocket — F. Hudson-Cox and Dr. J. Stokes (*Review*), 260.
Pharmacy, The mystery and romance of alchemy and—C. J. S. Thompson (*Review*), 48.
Philadelphia, Transactions of the College of Physicians of (*Review*), 266.
Photo-micrography — E. J. Spitta (*Review*), 72.
"Physicist"—Human nature: its principles and the principles of physiognomy (*Review*), 366.
Physiology, Kirkes' handbook of—Dr. W. D. Halliburton (*Review*), 354.
Physiology, Ludwig and modern—J. Burdon - Sanderson (*Review*), 250.
Physiology, Recent progress in—M. Foster (*Review*), 251.
Pick, T. P.—Surgery (*Review*), 61.
Platt, J. E.—A contribution to the surgery of fractures and dislocations of the upper extremity (*Review*), 367.
Plymouth Medical Society, 83.
Pneumonia, 141.
Poison romance and poison mysteries—C. J. S. Thompson (*Review*), 48.
Powell, Sir R. D.—On the principles which govern treatment in diseases and disorders of the heart (*Review*), 157.
Power, D'A.—How surgery became a profession in London (*Review*), 250.
Pregnancy, Extra-uterine — J. W. Taylor (*Review*), 69.
Prescriptions, Lessons on—W. H. Griffiths (*Review*), 170.
Preventive medicine, Some modern aspects of—Dr. D. S. Davies, 289.
Prichard, A. W.—Reminiscences of the Bristol Royal Infirmary, 193; Three abdominal cases presenting some unusual features, 313.
Prostate, Enlargement of the—Dr. C. M. Moullin (*Review*), 367.
Prostate, Treatment of enlarged, 237.

Psychiatry, Golden rules of—Dr. J. Shaw (*Review*), 262.
Psychology of sex, Studies in the—H. Ellis (*Review*), 50.
Pye's surgical handicraft (*Review*), 255.
Pyorrhœa alveolaris—J. Fitzgerald (*Review*), 259.

Ramsay, Dr. A. M.—Atlas of external diseases of the eye (*Review*), 360.
Ratzel, F.—The history of mankind (*Review*), 45.
Raynaud's disease—Dr. T. K. Monro (*Review*), 247.
Recollections of my life—Sir J. Fayrer (*Review*), 353.
Remedies, Rough notes on—Dr. W. Murray (*Review*), 260.
Remedies, The newer—Dr. V. Coblentz (*Review*), 260.
Renal cases—Dr. D. Newman (*Review*), 358.
Rheumatic disease of the cardiac muscle—Dr. T. Fisher, 16.
Rheumatism, 231.
Rheumatism—Dr. C. O. Hawthorne (*Review*), 366.
Rhinology, Report on—Dr. B. J. Baron, 348.
Rieder, Dr. H.—Atlas of urinary sediments (*Review*), 56.
Roberts, C.—Eastbourne as a health resort (*Review*), 262.
Robinson, Dr. T.—The diagnosis and treatment of eczema (*Review*), 370; The diagnosis and treatment of syphilis (*Review*), 171.
Robson, A. W. M.—Diseases of the gall-bladder and bile-ducts, including gall-stones (*Review*), 257.
Roentgen ray, Archives of the (*Review*), 273.
Roentgen ray—Skiagraphic atlas of fractures and dislocations—Dr. D. J. Mackintosh (*Review*), 68; The X-ray history of a fracture—J. Taylor, 214; Use of the X-ray in surgery, 344.
Rolleston, Dr. H. D.—The diseases and primary tumours of the thymus gland (*Review*), 167.
Rose and A. Carless, W.—A manual of surgery (*Review*), 367.
Roth, B.—The treatment of lateral curvature of the spine (*Review*), 168.

Saint Bartholomew's hospital reports (*Reviews*), 270.
Sanatoria for the poorer consumptives—Dr. L. A. Weatherly, 305.
Sanitation of British troops in India, The—Captain E. C. Freeman (*Review*), 167.
Sarcoma—W. R. Williams (*Review*), 165.
Sarcoma of the hard palate, Spindle-celled—W. J. Greer, 211.
Savill, Dr. T. D.—Clinical lectures on neurasthenia (*Review*), 168.
Schall's catalogue of electro-medical instruments (*Review*), 376.
Schoolboys' special immorality—Dr. M. C. Hime (*Review*), 263.
Schools, A code of rules for the prevention of infectious and contagious diseases in (*Review*), 168.
Schott methods of the treatment of chronic diseases of the heart, The—Dr. W. B. Thorne (*Review*), 255.
Science, Recent advances in—M. Foster (*Review*), 251.
Scraps, 95, 191, 287, 391.
Seitz, Dr. C.—Kurzgefasstes lehrbuch der kinderheilkunde (*Review*), 362.
Sex, Studies in the psychology of—H. Ellis (*Review*), 50.
Shaw, Dr. J.—Golden rules of psychiatry (*Review*), 262.
Shaw's manual of the vaccination law (*Review*), 261.
Shuttleworth, Dr. G. E.—Mentally-deficient children (*Review*), 368.
Sick, Preparations for the, 72, 172, 275, 377.
Skiagraphy—*see* Roentgen ray.
Smith, E. N.—Growing children: their clothes and deformity (*Review*), 169; Paralytic deformities of the lower extremities (*Review*), 359.
Smith, Dr. F. J.—Introduction to the outlines of the principles of differential diagnosis, with clinical memoranda (*Review*), 244.
Smith, Dr. F. W.—The natural waters of Harrogate (*Review*), 262.
Smith, G. M.—The spontaneous disappearance of a sarcomatous tumour, 30.
Smith, Dr. R. S.—Report on medicine, 141.
Smithsonian report (*Review*), 165.
Snell, S.—On the prevention of eye accidents occurring in trades (*Review*), 369.
Societies: Bristol Medico-Chirurgical, 75, 175, 381; Plymouth Medical, 83.

Spencer, Dr. W.—Spencer's disease: dermatitis multiformis exfoliativa (*Review*), 371.
Spencer, Dr. W. H—Consumption: its nature and treatment (*Review*), 254.
Spinal cord, Subacute combined degeneration of the, 340.
Spine, Lateral curvature of the—B. Roth (*Review*), 168.
Spitta, E. J.—Photo-micrography (*Review*), 72.
Stack, Dr. E. H. E.—A case of diffuse leontiasis ossea, 316.
Steven, Dr. J. L.—Lectures on clinical medicine (*Review*), 253.
Stokes, F. Hudson-Cox and Dr. J.—The pocket pharmacopœia (*Review*), 260.
Stokes, W.—William Stokes: his life and work (1804–1878) (*Review*), 49.
Stomach and duodenum, Ulcer of the—Drs. S. and W. S. Fenwick (*Review*), 357.
Stomach, The diseases of the—Drs. W. W. van Valzah and J. D. Nisbet (*Review*, 58.
Stone, Selected papers on—R. Harrison (*Review*), 161.
Stonham, C.—A manual of surgery (*Review*), 62.
Suprarenal extract, Secondary hemorrhage after the use of, 349.
Surgery—T. P. Pick (*Review*), 61; W. Rose and A. Carless (*Review*), 367; C. Stonham (*Review*), 62.
Surgery became a profession in London, How—D'A. Power (*Review*), 250.
Surgery, Lord Lister and—Dr. R. Turner (*Review*), 250.
Surgery, Reports on—T. Carwardine, 236; C. A. Morton, 146; Dr. J. Swain, 36, 343.
Surgical handicraft, Pye's (*Review*), 255.
Surgical treatment, A manual of—Drs. W. W. Cheyne and F. F. Burghard (*Review*), 247.
Swain, Dr. J.—A retrospect of a second series of fifty consecutive intra-abdominal operations, 103; Reports on surgery, 36, 343.
Swayne, Obituary notice of Mr. S. H., 390.
Swayne, Dr. W. C.—Abdominal hysterectomy for fibroids of the uterus, with retro-peritoneal treatment of the stump, 123; Report on obstetrics, 151.
Swedish medical gymnastics—E. Christofferson, 134.
Sydney, The sanitary value of the operations of the Board in reducing and avoiding the mortality from typhoid fever in the metropolis of (*Review*), 272.
Symes, Dr. J. O.—Non-bacillary croup, 25; The Pasteurisation of milk, 275.
Syphilis and the venereal diseases, Atlas of—Dr. F. Mracek (*Review*), 60.
Syphilis, The diagnosis and treatment of—Dr. T. Robinson (*Review*), 171.

Taylor, J.—The X-ray history of a fracture, 214.
Taylor, J. W.—Extra-uterine pregnancy (*Review*), 69.
Therapeutics, Materia medica and—Dr. J. M. Bruce (*Review*), 260.
Thermal procedures, Notes on some—Dr. C. J. Whitby, 1.
Thompson, C. J. S.—Poison romance and poison mysteries (*Review*), 48; The mystery and romance of alchemy and pharmacy (*Review*), 48.
Thompson, Sir H.—Modern cremation (*Review*), 66.
Thomson, Dr. H. C.—An introduction to diseases of the nervous system (*Review*), 245.
Thomson, Dr. StC.—The cerebro-spinal fluid; its spontaneous escape from the nose (*Review*), 162.
Thorne, Dr. W. B.—The Schott methods of the treatment of chronic diseases of the heart (*Review*), 255.
Throat and nose and their diseases, The—L. Browne (*Review*), 164.
Throat, nose and ear, Nursing in diseases of the—M. Yearsley (*Review*), 71.
Thymus gland, The diseases and primary tumours of the—Dr. H. D. Rolleston (*Review*), 167.
Tilsley, Obituary notice of Mr. J., 390.
Tirard, Dr. N.—Albuminuria and Bright's disease (*Review*), 158; A text-book of medical treatment (*Review*), 355.
Trachéo-thyrotomie dans le cancer du larynx, De la—Dr. E. J. Moure (*Review*), 258.
Trades, Eye accidents occurring in—S. Snell (*Review*), 369.
Treatment, Medical—Dr. J. M. Bruce (*Review*), 356; Dr. N. Tirard (*Review*), 355.

Treves, F.—Intestinal obstruction (*Review*), 256.
Troops in India, The sanitation of British—Captain E. C. Freeman (*Review*), 167.
Tropical diseases—Dr. P. Manson (*Review*), 365.
Tuberculosis—Dr. A. Hillier (*Review*), 253.
Tuchmann, M.—The exploration of the urethra and bladder (*Review*), 63.
Tumour, The spontaneous disappearance of a sarcomatous—G. M. Smith, 30.
Turner, Dr. R.—Lord Lister and surgery (*Review*), 250.
Typhoid fever, 7, 229; Diet in, 33.
Typhoid fever in Sydney (*Review*), 272.

Ulcer of the stomach and duodenum—Drs. S. and W. S. Fenwick (*Review*), 357.
Urethra and bladder, The exploration of the—M. Tuchmann (*Review*), 63.
Urethra, Vascular caruncle of the—Dr. A. E. A. Lawrence, 23.
Urinary sediments, Atlas of—Dr. H. Rieder (*Review*), 56.
Uterine douche, Use of the intra-, 151.

Vaccination—Dr. S. M. Copeman (*Review*), 65.
Vaccination law, Shaw's manual of the (*Review*), 261.
Valzah and J. D. Nisbet, Drs. W. W. van—The diseases of the stomach (*Review*), 58.
Venereal diseases, Atlas of—Dr. F. Mracek (*Review*), 60.
Vice and insanity—Dr. G. R. Wilson (*Review*), 245.
Viscera, On the relation of the nervous system to disease and disorder in the—Dr. A. Morison, (*Review*), 54.
Vital statistics—Dr. A. Newsholme (*Review*), 71.

Waldo, Dr. H.—Report on dermatology, 240.
Walker, Dr. Jane H.—Open-air treatment of consumption—seven years' experience in England (*Review*), 169.
Walker, Dr. N.—An introduction to dermatology (*Review*), 163.
Ward work and nursing, Surgical—Dr. A. Miles (*Review*), 371.
Watson, Dr. C. [Ed.]—Encyclopædia medica (*Reviews*), 55, 156.
Watson, Dr. J. K.—A handbook for nurses (*Review*), 162.
Weatherly, Dr. L. A.—A plea for sanatoria for the poorer consumptives, 305.
Whitby, Dr. C. J.—Notes on some thermal, hydro-thermal, electric and hydro-electric procedures, and the indications for their use, 1.
Whiteford, C. H.—One thousand consecutive inductions of general anæsthesia, 217; The technique of Halsted's operation for cancer of the breast, 336.
Whitelegge, Dr. B. A.—Hygiene and public health (*Review*), 169.
Williams, Dr. P. W.—Note on a pre-exanthematous sign of measles, 139.
Williams, W. R.—Cancer; Sarcoma (*Reviews*), 165.
Wilson, Major E. M.—Notes on malaria in connection with meteorological conditions at Sierra Leone (*Review*), 255.
Wilson, Dr. G. R.—Clinical studies in vice and in insanity (*Review*), 245.

X-rays—*see* Roentgen ray.
Xeroform (*Review*), 261.

Yearsley, M.—Nursing in diseases of the throat, nose, and ear (*Review*), 71.

J. W. Arrowsmith, Printer, Quay Street, Bristol.

